# Editorial: Assessment and monitoring of human movement, volume II

**DOI:** 10.3389/fphys.2026.1995658

**Published:** 2026-09-29

**Authors:** Daniel López-Plaza, Giuseppe Annino, Cristian Romagnoli

**Affiliations:** 1 Entrenamiento, Actividad Física y Rendimiento Deportivo (ENFYRED) Research Group, Faculty of Health and Sports Sciences, University of Zaragoza, Huesca, Spain; 2 Faculty of Education, University of Zaragoza, Zaragoza, Spain; 3 Human Performance Laboratory, Centre of Space Bio-Medicine, Department of Systems Medi-cine, University of Rome Tor Vergata, Rome, Italy; 4 Human Performance, Sport Training, Health Education Laboratory, Department of Human Science and Promotion of Quality of Life, San Raffaele Open University, Rome, Italy

**Keywords:** human movement monitoring, injury prevention, markerless motion capture, neuromuscular performance, sports biomechanics, training load, wearable sensors (IMU)

## Evolution and current state of human movement assessment and monitoring

The quantitative study of human movement has evolved from classical observational mechanics into a sophisticated, sensor-driven scientific discipline (Edriss et al., 2024; Innocenti, 2018). While early biomechanical principles were framed through anatomical observations, the transition toward technical and objective measurements became essential in the nineteenth century. This shift was marked by the chronophotography of Marey and Muybridge (Marey, 1885, 1888; Muybridge, 1887) and early kinetic devices, able to measure joint amplitude and force variations (Amar, 1916), eventually culminating in the introduction of piezoelectric force platforms (Baker, 2007; Bestwick-Stevenson et al., 2022) and others kinematic device as conductive mat (Bosco et al., 1983) and linear encoder (Bosco et al., 1995).

In recent decades, the rapid convergence of micro-electro-mechanical systems (MEMS), the Internet of Things (IoT), wireless telemetry, and smart textiles has revolutionized movement assessment (Edriss et al., 2024). The development of specialized instrumentation, such as inertial measurement units (IMUs), global navigation satellite systems (GNSS), ultra-wideband (UWB) tracking, portable gas analyzers, and surface electromyography, offers profound technological and practical advantages across clinical, athletic and educational spectrums (Bourdon et al., 2017; Camomilla et al., 2018). Fundamentally, these specialized instruments replace subjective visual appraisal with granular, highly reproducible kinematic and kinetic metrics. This precision eliminates evaluator bias and enables accurate longitudinal tracking of physical development, technical mastery, and motor recovery (Ancillao, 2018). Furthermore, by concurrently capturing external mechanical demands (e.g., velocity, accelerations, and linear load) and internal physiological responses (e.g., heart rate variability and respiratory parameters), practitioners and sport coaches can optimize training periodization, identify premature fatigue, and guide safe return-to-play protocols (Halson, 2014; Impellizzeri et al., 2019; Taberner et al., 2019). Additionally, modern sensor technologies deliver immediate biofeedback during movement execution, enabling coaches and clinicians to make precise real-time adjustments (Edriss et al., 2024; Zanela et al., 2022; Ziegler et al., 2026). Finally, the high ecological validity of wearable systems allows for non-invasive data collection in naturalistic environments, supporting early detection of gait anomalies, objective monitoring of disease progression and evidence-based physical education interventions (Doherty et al., 2016; Edriss et al., 2024).

Today, integrating multi-sensor fusion with artificial intelligence and machine learning algorithms is propelling human movement monitoring into a predictive, highly personalized era, fundamentally reshaping performance optimization, injury prevention and clinical health management.

## Overview of the contributions

This Research Topic compiles 14 articles, comprising original research, systematic reviews and mini-reviews, that showcase the latest methodological, technological, and practical advancements in movement assessment. Collectively, these contributions span four interconnected thematic axes that reflect the modern landscape of sports biomechanics, clinical rehabilitation and training load monitoring.

### Technical and kinematic optimization in elite sport.

High-precision biomechanical profiling has proven essential for decoding movement mechanics and refining discipline-specific performance. In speed skating, applying Classification and Regression Tree (CART) machine learning to 3D kinematics and kinetic force plate data enabled researchers to identify critical inter-limb asymmetry thresholds directly linked to split skating times (Jin et al.). In track and field, kinematic analyses comparing elite Chinese and international female throwers highlighted the crucial role of release velocity, double-support mechanics, and trunk–pelvis coupling in hammer throw performance (Liu et al.). Similarly, three-dimensional motion capture combined with ground reaction force analysis defined the spatial-temporal and force-impulse demands of the skeleton start, establishing empirical guidelines for specialized push simulations and sprint acceleration (Hao and Kong). In trampolining, electromyography and plantar pressure assessments during the net contact phase revealed sex-specific lower-limb dynamics and balance strategies among elite athletes, emphasizing the necessity of targeted unilateral training (Liu et al.). Finally, anthropometric and maturation predictive equations were developed for young kayakers, providing an objective, scientific framework to scale paddle dimensions across developmental age categories (Fernandes et al.).

### Neuromuscular profiling, inter-limb asymmetry and injury risk

Neuromuscular evaluations and functional asymmetry analyses remain central to optimizing conditioning and mitigating injury risk across various populations. An evaluation of inter-limb differences in balance, jumping, and isokinetic strength among elite adolescent basketball players demonstrated that while large strength asymmetries (>20%) exist, they do not significantly differ between sexes, reframing conventional assumptions regarding female injury risk (Lyu et al.). Investigating isokinetic knee strength and agility performance in elite Wushu Changquan athletes established foundational parameters for neuromuscular coordination and lower-limb balance in martial arts science (Zhiyong et al.). From a clinical perspective, a novel cross-line laser balance tool was validated against gold-standard force plates, demonstrating high agreement and proving to be a cost-effective solution for objective balance assessment in individuals with chronic ankle instability (Swails et al.).

### Training load, fatigue dynamics and field testing protocols

Comprehensive monitoring of physical performance and physiological responses is crucial for effective load management and intervention programming. Evaluating load-velocity relationships and sport-specific skill performance in female softball players demonstrated that while mechanical metrics decline post-exercise, integrated biochemical and perceptual markers remain essential for capturing subtle fatigue responses (Zhang et al.). In field testing, a re-evaluation of the 30–15 Intermittent Fitness Test in collegiate soccer players confirmed its high test-retest reliability, while demonstrating that population-specific prediction equations significantly reduce error when estimating maximal oxygen consumption VO_2max_ compared to generalized models (Cheng et al.). Furthermore, a systematic review and meta-analysis of randomized controlled trials demonstrated that plyometric jump training yields significant improvements in jumping, agility, and kicking power in female soccer players, with particularly pronounced effects observed in professional athletes (Hernandez-Martinez et al.). Addressing team sports load management, a comprehensive review synthesized internal and external load indicators in rugby, emphasizing the critical need for multi-sensor algorithms and individualized monitoring frameworks to manage fatigue and reduce injury risk (Lv et al.).

### Emerging technologies, sensor fusion, and non-invasive assessment

Modern advancements in optical sensors and non-invasive technologies are expanding the scope and accessibility of movement analysis. A mini-review evaluated commercial RGB/depth cameras and AI-based pose estimation frameworks, highlighting the rapid evolution toward affordable, non-invasive 2D-to-3D markerless motion analysis for both clinical and sporting environments (Edriss et al.). Additionally, high-sampling-rate thermal imaging combined with chest temperature entropy analysis was introduced as an innovative, non-invasive method to monitor physiological load and excess post-exercise oxygen consumption (EPOC) during incremental cycling (Cui et al.).

## Conclusion and future perspectives

The collective body of evidence compiled in this *Research Topic* underscores a profound paradigm shift in human movement assessment, bridging the historical gap between laboratory-based biomechanics and real-world ecological applications. By integrating advanced wearable sensors, machine learning analytics, markerless pose estimation, and sport-specific field protocols, these 14 contributions demonstrate how objective, high-resolution monitoring enhances athletic performance, optimizes injury mitigation, and refines clinical rehabilitation across diverse populations worldwide. Beyond their specific findings, the global impact of these studies lies in the democratization and personalization of movement science. Moving away from isolated, one-size-fits-all evaluations, this volume establishes actionable, evidence-based frameworks that adapt to age, sex, athletic tier and clinical status, ranging from customized equipment scaling in youth sports to multi-modal training load management in elite competition.

In the future, the trajectory of human movement monitoring might be defined by the seamless convergence of multi-sensor fusion, real-time automated biofeedback and predictive artificial intelligence. As these non-invasive technologies mature, they promise to deliver unprecedented insights into human function, fostering a more precise and globally accessible era of movement analysis in sports. We express our sincere gratitude to all contributing authors, expert reviewers and the editorial team whose dedication made this volume a meaningful contribution to the scientific community.
